# Parasites in the Fossil Record: A Cretaceous Fauna with Isopod-Infested Decapod Crustaceans, Infestation Patterns through Time, and a New Ichnotaxon

**DOI:** 10.1371/journal.pone.0092551

**Published:** 2014-03-25

**Authors:** Adiël A. Klompmaker, Pedro Artal, Barry W. M. van Bakel, René H. B. Fraaije, John W. M. Jagt

**Affiliations:** 1 Florida Museum of Natural History, University of Florida, Gainesville, Florida, United States of America; 2 Department of Geology, Kent State University, Kent, Ohio, United States of America; 3 Museo Geológico del Seminario de Barcelona, Barcelona, Spain; 4 Oertijdmuseum De Groene Poort, Boxtel, The Netherlands; 5 Naturalis Biodiversity Center, Leiden, The Netherlands; 6 Natuurhistorisch Museum Maastricht, Maastricht, The Netherlands; University of Western Ontario, Canada

## Abstract

Parasites are common in modern ecosystems and are also known from the fossil record. One of the best preserved and easily recognisable examples of parasitism in the fossil record concerns isopod-induced swellings in the branchial chamber of marine decapod crustaceans. However, very limited quantitative data on the variability of infestation percentages at the species, genus, and family levels are available. Here we provide this type of data for a mid-Cretaceous (upper Lower Cretaceous, upper Albian) reef setting at Koskobilo, northern Spain, on the basis of 874 specimens of anomurans and brachyurans. Thirty-seven specimens (4.2%), arranged in ten species, are infested. Anomurans are more heavily infested than brachyurans, variability can be high within genera, and a relationship may exist between the number of specimens and infestation percentage per taxon, possibly suggesting host-specificity. We have also investigated quantitative patterns of infestation through geological time based on 88 infested species (25 anomurans, 55 brachyurans, seven lobsters, and one shrimp), to show that the highest number of infested species can be found in the Late Jurassic, also when corrected for the unequal duration of epochs. The same Late Jurassic peak is observed for the percentage of infested decapod species per epoch. This acme is caused entirely by infested anomurans and brachyurans. Biases (taphonomic and otherwise) and causes of variability with regard to the Koskobilo assemblage and infestation patterns through time are discussed. Finally, a new ichnogenus and -species, *Kanthyloma crusta*, are erected to accommodate such swellings or embedment structures (bioclaustrations).

## Introduction

Parasites are common in modern ecosystems (e.g., [1], [2]) and are also known from the terrestrial and marine fossil record (e.g., [3]–[6]). Parasites are ecologically and evolutionarily important because they influence food webs and can lead to co-evolution of host and parasite. Convincing evidence of parasites in marine invertebrates is known since the Ordovician, yet the number of parasitic associations is often lower than ten per geologic period [5], at least in part related to insufficient preservation and recognition.

**Figure 1:**
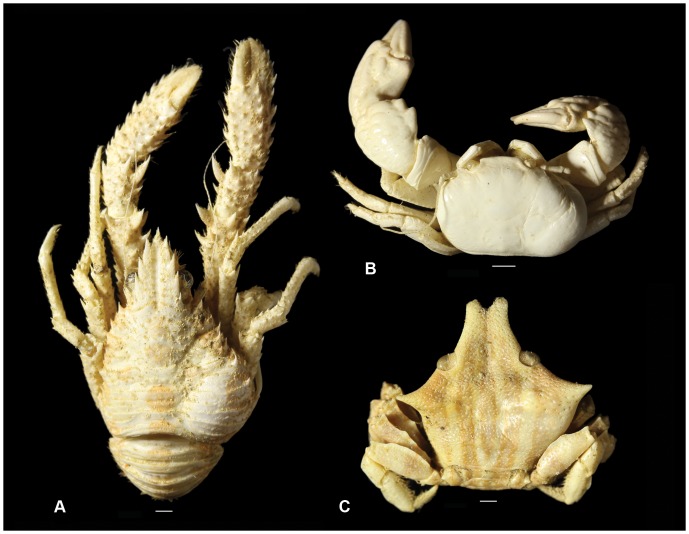
Examples of isopod-infested decapods from the modern environment. A, *Galathea* sp., Camiguin, Philippines (MAB k. 3297); B, *Pachycheles garciaensis* [122], Camiguin, Philippines (MAB k. 3298); C, *Eumedonus zebra* [123], Camiguin, Philippines (MAB k. 3299). Scale bars are 1.0 mm wide.

The order Isopoda comprises a diverse array of generally small crustaceans, with over 10,000 extant species known from terrestrial (i.e., woodlice and pill bugs), and fresh- and ocean water settings [7]. Swellings of parasitic isopods that use other crustaceans as hosts (i.e., epicarideans) are found commonly in the fossil record, are easily recognised, and preserve relatively well. Extant epicarideans include the superfamilies Bopyroidea and Cryptoniscoidea [8] (Fig. 1). The cryptoniscoideans are mentioned to be endoparasitic to crustaceans including some decapods [8], but no body or other fossil evidence is known. Extant dajid cryptoniscoids are ectoparasitic and found on several hosts (euphausiids, mysids and shrimps) [8]. Extant bopyroids are assigned to three families, the Bopyridae, Ionidae and Entoniscidae [9]. Entoniscids are endoparasitic, whereas members of the two other families are mostly ectoparasitic, inclusive of occurrence under the cuticle on the gills [8]. Bopyrids and ionids have been recorded from a variety of extant decapod crustaceans including shrimps, Anomura, and Brachyura [8], [10]. These ectoparasitic epicarideans cause the formation of swellings (‘cysts’) or deformations of the cuticle of their hosts, often in the branchial region of decapod crustaceans. New cuticle bulges around the parasite after each moulting event and may leave space for the female isopod (and often the accompanying, smaller male) to grow, perhaps resulting in less damage to the gills than otherwise would have been inflicted [11]; thus, the isopod grows along with its decapod host (e.g., [12]). Importantly, similarly shaped swellings have also been documented from the fossil record (e.g., [13]–[20]) and arguably represent one of the most obvious examples of parasitism in the fossil record. These have generally been assumed to be of bopyrid origin. The appearance as swellings does not allow identification at lower taxonomic ranks and, as such, has little or nothing to add to the diversity of extinct isopods. The stratigraphically oldest swellings on record are from the Jurassic, i.e., Oxfordian (Late Jurassic, 163.5–157.3 Ma) for Anomura and Brachyura and ?Toarcian (Lower Jurassic; 182.7–174.1 Ma) for lobsters, but no infested specimens of fossil shrimps were known [18] until very recently [182].

Unfortunately, there are no records of isopod remains from within such swellings. However, most of the authors listed above (i.e., [14]–[16], [19]) did ascribe them to infestation by bopyrids, with reference to extant examples. However, Wienberg Rasmussen *et al*. [18] suggested to use the term ‘bopyriform swelling’ instead, in view of the fact that other epicaridean genera are known to infest modern decapod crustaceans and that carapaces with other deformities are on record, although they provided no details in support of this. However, Shields & Kuris [21] reported on a lateral inflation in the left branchial region of the extant brachyuran *Hemigrapsus nudus* ([22]) caused by an isopod of the family Entoniscidae. The assumption made by many authors is that members of the family Bopyridae have been around since the Jurassic and have been infesting the same groups of decapod crustaceans and in the same general carapace area since that time. Obviously, such a hypothesis cannot be tested unless isopods are actually found within their host, which requires exquisite preservation. We concur with Wienberg Rasmussen *et al*. [18] that it cannot be considered a fact that these swellings have indeed all been caused by bopyrid isopods. Instead, the great majority of these swellings are most likely the result of embedment by some group, or several groups, within the order Isopoda. Thus, they can be considered to represent possible trace fossils of a bioclaustration signature.

Very limited quantitative data with regard to the occurrence of such swellings in the fossil record are available. Most data refer to percentages for entire decapod crustacean assemblages. On the basis of 3,000 specimens, Bachmayer [23] noted that 2% of Tithonian (Late Jurassic, 152.1–145.0 Ma) anomurans (galatheoids) and brachyurans (‘prosoponiden’) from Ernstbrunn (Austria) were affected, while Houša [15] computed 3.82% for Tithonian assemblages from Štramberk (Czech Republic), based on 890 specimens. For the Oxfordian of Poland, Radwański [16] noted that seven out of more than 500 ‘prosoponid’ brachyurans (i.e., <1.4%) revealed a swelling. These comprised the species *Pithonoton marginatum* [24] (5 infested specimens), and one specimen each of *Eodromites rostratus* ([25]) and *Planoprosopon heydeni* ([26]). Wienberg Rasmussen *et al*. [18] supplied some species-level data for the Maastrichtian (Late Cretaceous, 72.1–66.0 Ma) of West Greenland, as follows: 73/1,295 ( = 5.64%) specimens of the raninoid *Macroacaena rosenkrantzi* ([27]) were affected, whereas the contemporary congener *M. succedana* ([27]) was free from infestation, presumably based on 193 specimens [27]. The co-occurring lobster *Mecochirus rostratus* [27], yielded a low percentage (2/2,163, ∼0.1%).

The goals of the present paper are to record swellings in the branchial chamber from a variety of anomuran and brachyuran decapods from the Lower Cretaceous (upper Albian) of northern Spain, to address biases and quantify infestation patterns through time, and to erect and describe a new ichnogenus and -species. Finally, since previous studies of such ‘cysts’ suffered from limited quantification, we suggest a systematic methodology of collecting and counting along a range of systematic ranks, which has not been done previously.

## Materials and Methods

The material studied was collected from the Aldoirar patch reef as exposed at the Koskobilo quarry (42°52′56″N/2°11′59″W) in northern Spain, 2 km southwest of Alsasua (see [28]). The reef-associated limestones from the Eguino Formation (Albeniz Unit) are of mid-Cretaceous (late Early Cretaceous, late Albian) age [29]. The taxonomy and paleoecology of decapod crustaceans from this locality have recently been studied in detail [28]–[38]. Decapod crustaceans were collected from this locality during the summers of 2008–2012; these form the basis for the present study. In total, 874 anomuran and brachyuran specimens, assigned to 30 species, 21 genera and 14 families were studied for swellings. A bias towards certain decapod groups or infested decapods was circumvented by collecting all specimens. In order to quantify the percentage of swellings per taxon, specimens with both branchial sides visible to the extent that the presence or absence of a swelling could be determined for both branchial sides, were selected. The percentage of specimens with swellings was studied subsequently at the species, genus, and family levels. It was also noted whether the swelling occurred in the left or right branchial side. The Cementos Portland Valderrivas Company (Olazti, Navarra), on behalf of Anna Hernández, gave permission for fieldwork in their Koskobilo quarry. All necessary permits were obtained for the described study, which complied with all relevant regulations.

To study infestation patterns through geological time, the number of infested species per post-Triassic epoch is compiled and standardised per 20 Myr for each epoch. The January 2013 geological time scale of the International Commission on Stratigraphy was used to obtain the duration of each epoch. Periods after the Triassic were chosen because these swellings are unknown from pre-Jurassic decapods. Since this measure could be influenced by a variable number of species present per epoch, the percentage of species infested per epoch was calculated from the Early Jurassic – Late Cretaceous only because most variation in the number of infested species occurred within this time frame as suggested by previous research [18] and results herein. Decapod species diversity also fluctuated greatly in the Jurassic and Cretaceous [39]. Species recorded solely from an epoch boundary interval have not been included. Both measures were done for all Decapoda as well as for the decapod subgroups Brachyura, galatheoid anomurans, and lobsters (infraorders Achelata, Astacidea, Glypheidea, and Polychelidea) since swellings are only known from these decapod groups. Additionally, a possible relationship between the number of specimens and the percentages of infestation is studied at the species, genus, and family level.

Specimen numbers preceded by MAB are stored in Oertijdmuseum De Groene Poort, Boxtel, The Netherlands. Those preceded by MGSB are deposited in Museo Geológico del Seminario de Barcelona, Barcelona, Spain.

### Nomenclatural Acts

The electronic edition of this article conforms to the requirements of the amended International Code of Zoological Nomenclature, and hence the new names contained herein are available under that Code from the electronic edition of this article. This published work and the nomenclatural acts it contains have been registered in ZooBank, the online registration system for the ICZN. The ZooBank LSIDs (Life Science Identifiers) can be resolved and the associated information viewed through any standard web browser by appending the LSID to the prefix “http://zoobank.org/”. The LSID for this publication is: urn:lsid:zoobank.org:pub:9EAA81B7-42F3-406F-9AE7-23989AA15238. The electronic edition of this work was published in a journal with an ISSN, and has been archived and is available from the following digital repositories: PubMed Central, LOCKSS.

## Results

**Figure 2:**
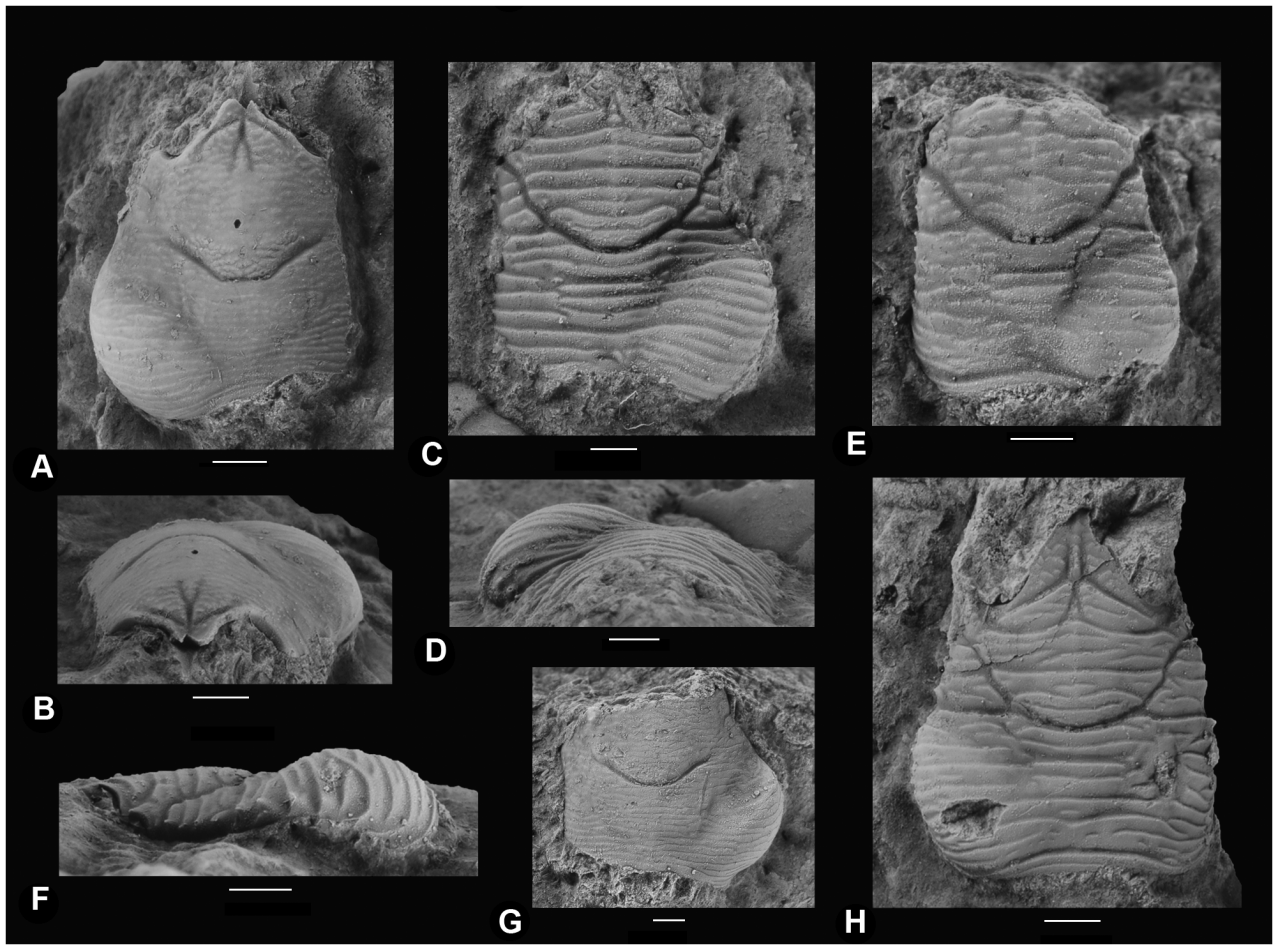
Carapaces of late Albian anomurans from Koskobilo (Spain) showing swellings (*Kanthyloma crusta*) in the branchial regions. *Paragalathea ruizi* (MAB k. 3008, with paratype of *Kanthyloma crusta*, MAB k. 3008-i) in dorsal (A) and frontal view (B); *Eomunidopsis navarrensis* (MGSB78339, with holotype of *Kanthyloma crusta,* MGSB78340) in dorsal (C) and frontal view (D); E, *Eomunidopsis aldoirarensis* (MAB k. 2981+ ichnofossil MAB k. 2981-i) in dorsal view; F, *Eomunidopsis navarrensis* (MAB k. 2982+ ichnofossil MAB k. 2982-i) in left-lateral view; G, *Paragalathea ruizi* (MAB k. 3189+ ichnofossil MAB k. 3189-i) in dorsal view; and H, *Eomunidopsis navarrensis* (MAB k. 2644+ ichnofossil MAB k. 2644-i) in dorsal view. Scale bars are 1.0 mm wide.

**Figure 3:**
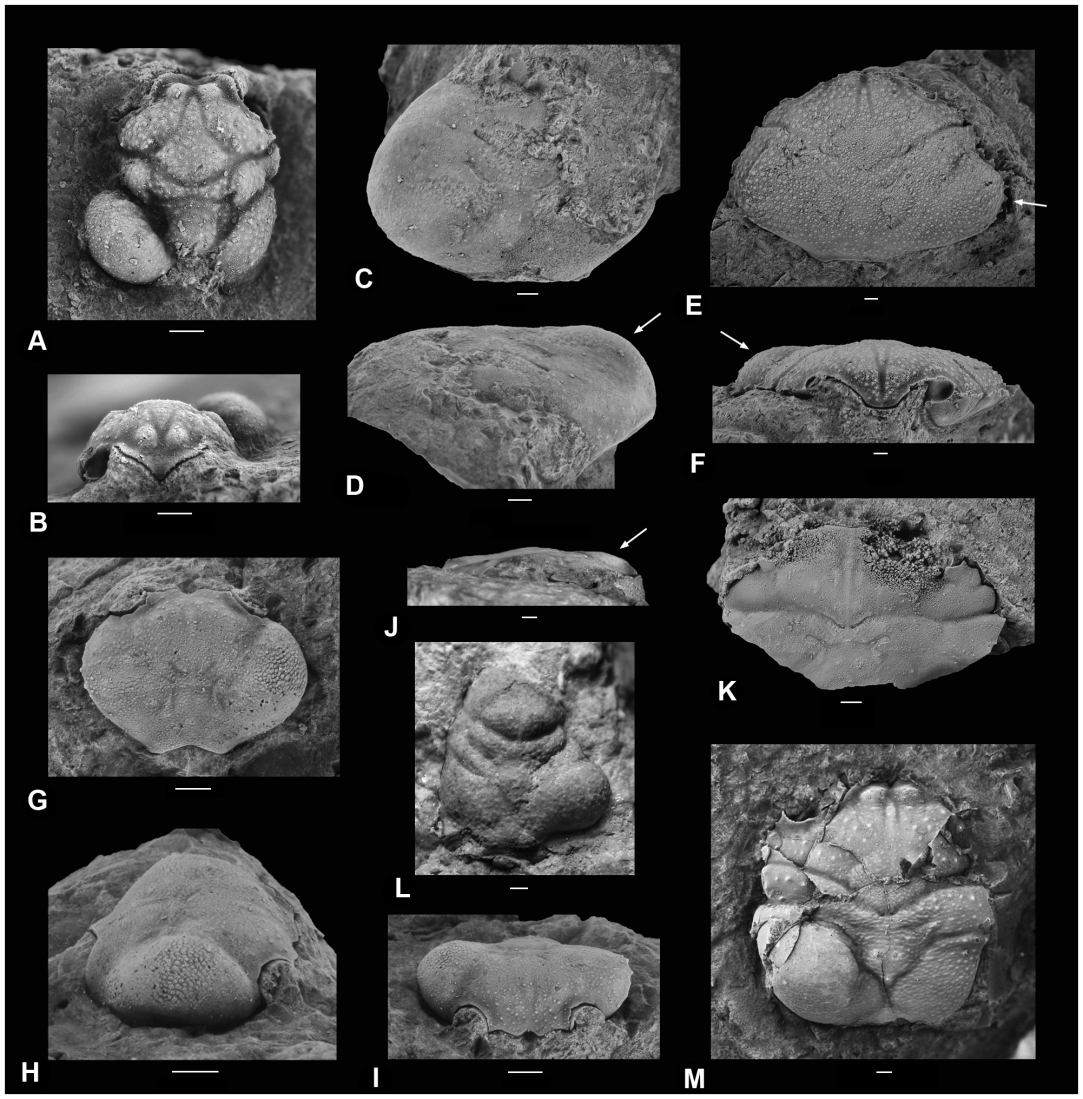
Carapaces of late Albian brachyurans from Koskobilo (Spain) showing swellings (*Kanthyloma crusta*) in the branchial regions. *Acareprosopon bouvieri* (MAB k. 3300+ ichnofossil MAB k. 3300-i) in dorsal (A) and frontal view (B); *Graptocarcinus texanus* (MAB k. 3174+ ichnofossil MAB k. 3174-i) in dorsal (C) and frontal view (D); *Distefania renefraaijei* (MAB k. 2601, paratype *D. renefraaijei* with paratype of *Kanthyloma crusta*, MAB k. 2601-i) in dorsal (E) and frontal view (F); *Caloxanthus paraornatus* (MAB k. 3177, with paratype of *Kanthyloma crusta*, MAB k. 3177-i) in dorsal (G), right-lateral (H), and frontal view (I); *Faksecarcinus* cf. *F. koskobiloensis* (MAB k. 3149+ ichnofossil MAB k. 3149-i) in frontal (J) and dorsal view (K); *Viaia robusta* (MGSB78508+ ichnofossil MGSB78509) in dorsal view (L); and *Goniodromites laevis* (MAB k. 3301+ ichnofossil MAB k. 3301-i) in dorsal view. E, F modified from Klompmaker *et al.* [34]; K modified from Klompmaker *et al.* [28]. Scale bars are 1.0 mm wide.

A stepwise decrease in the percentage of infestation is observed from the family to the species level: 7/14 (50%) of the families, 9/21 (43%) of the genera, and 10/30 (33%) of the species present in the sample from Koskosbilo were infested. At the specimen level, 37 of 874 decapod crustaceans (4.2%) showed a swelling at one of the branchial chambers (Table 1); no examples were found in which both branchial sides were infested. For anomurans only, 29 of 386 specimens (7.5%) showed a swelling (Fig. 2 for examples), whereas only 8 of 488 brachyuran specimens (1.6%) were infested (Fig. 3 for examples). At the family level for those represented by at least 30 specimens, galatheid specimens are most affected (29/373, 7.8%), whereas brachyuran families show distinctly lower infestation percentages (<2.0%). A similar pattern appears at the genus level and species level: the galatheoids *Eomunidopsis* (11.2%) and *Paragalathea* (3.5%) are more heavily infested than brachyuran genera (<2.0%), which is mainly caused by infestation in abundantly present specimens of *Eomunidopsis navarrensis* (12.1%) and *Paragalathea ruizi* (3.7%). Some infestation percentages in decapod species are high (*E. aldoirarensis* and *Faksecarcinus* (cf.) *koskobiloensis*), but those are based on a limited number of specimens so far. There is no statistical preference for either the left or right branchial side for infestation on the assemblage level (χ^2^ = 0.03, p = 0.99) and for the most commonly infested species, *E. navarrensis* (χ^2^ = 0.05, p = 0.98) (Table 1). In contrast, preference for infestation in the left and right side was proposed and discussed previously for fossil and extant decapods, especially galatheoids (e.g., [12], [14], [18], [40]).

**Table 1:** The total number of studied specimens per species from the late Albian Koskobilo fauna, the number of specimens with a swelling, the percentage of infestation, and the number of infested specimens per branchial side.

Anomuran (A) orbrachyuran (B) family	Genus and species	Specimensstudied	Specimenswith a swelling	Percentage	Swelling in leftbranchial side	Swelling in rightbranchial side
(A) Galatheidae	*Eomunidopsis aldoirarensis* [32]	17	3	17.6	2	1
(A) Galatheidae	*E. navarrensis* ([127])	174	21	12.1	11	10
(A) Galatheidae	*E. orobensis* ([128])	24	0	0.0		
(A) Galatheidae	*Hispanigalathea pseudolaevis* [32]	15	0	0.0		
(A) Galatheidae	*H. tuberosa* [32]	1	0	0.0		
(A) Galatheidae	*Paragalathea multisquamata* [129]	2	0	0.0		
(A) Galatheidae	*P. ruizi* ([127])	136	5	3.7	1	4
(A) Galatheidae	*P. straeleni* ([128])	4	0	0.0		
(A) Munidopsidae	*Nykteripteryx rostrata* [32]	3	0	0.0		
(A) Gastrodoridae	*Gastrodorus cretahispanicus* [31]	10	0	0.0		
(B) ?Macropipidae	*Faksecarcinus* (cf.) *koskobiloensis* ([28])	10	2	20.0	2	
(B) Dynomenidae	*Graptocarcinus texanus* [130]	59	1	1.7	1	
(B) Etyidae	*Etyxanthosia fossa* ([131])	2	0	0.0		
(B) Feldmanniidae	*Caloxanthus paraornatus* [28]	59	1	1.7		1
(B) Goniodromitidae	*Distefania incerta* ([132])	69	0	0.0		
(B) Goniodromitidae	*D. renefraaijei* [34]	13	1	7.7		1
(B) Goniodromitidae	*Eodromites grandis* ([26])	23	0	0.0		
(B) Goniodromitidae	*Goniodromites laevis* ([127])	119	1	0.8	1	
(B) Goniodromitidae	*Laeviprosopon crassum* [29]	4	0	0.0		
(B) Goniodromitidae	*L. edoi* [29]	3	0	0.0		
(B) Goniodromitidae	*L. hispanicum* [29]	2	0	0.0		
(B) Goniodromitidae	*L. planum* [29]	2	0	0.0		
(B) Goniodromitidae	*Navarradromites pedroartali* [34]	11	0	0.0		
(B) Prosopidae	*Acareprosopon bouvieri* ([133])	70	1	1.4	1	
(B) Prosopidae	*Rathbunopon obesum* ([133])	15	0	0.0		
(B) Homolidae	*Navarrahomola hispanica* [35]	2	0	0.0		
(B) Longodromitidae	*Navarrara betsiei* [29]	4	0	0.0		
(B) Necrocarcinidae	*Glyptodynomene alsasuensis* [133]	4	0	0.0		
(B) Torynommidae	*Albenizus minutus* [29]	3	0	0.0		
(B) Viaiidae	*Viaia robusta* [35]	14	1	7.1		1
	***total***	**874**	**37**	**4.2**	**19**	**18**

Paguroids [30], [36] are not listed as their branchial chambers are not preserved typically; the rare priscinachid majoids [29] did not preserve both branchial sides.

**Figure 4:**
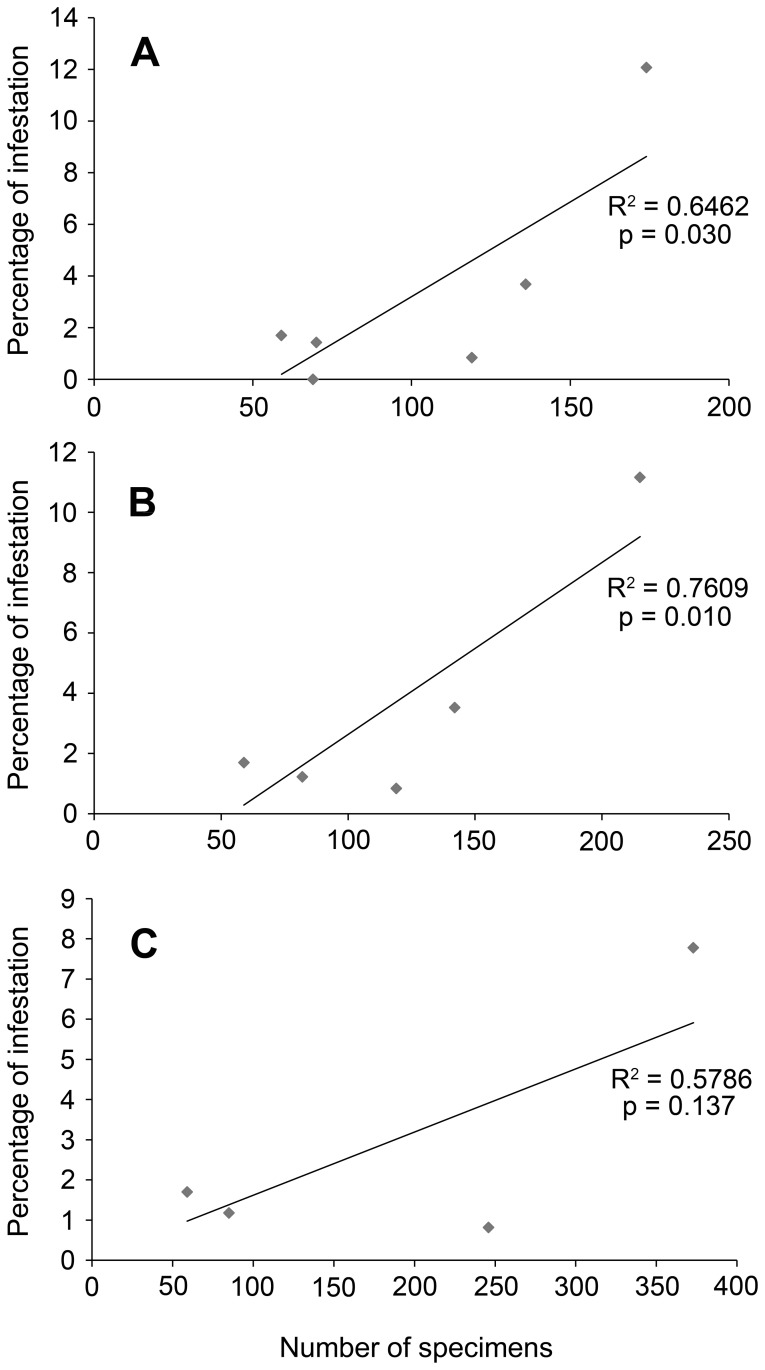
The number of specimens *vs* infestation percentage at different taxonomic levels. A, Species level; B, Genus level; C, Family level. Only those taxa with at least 30 specimens are included.

**Figure 5:**
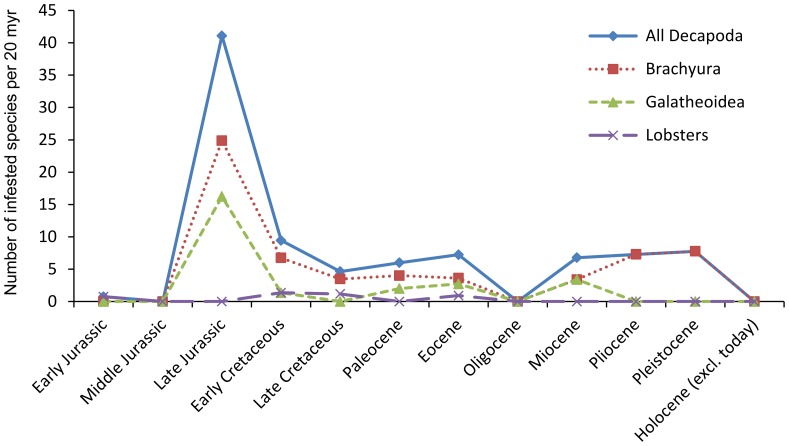
The number of infested marine decapod, brachyuran, galatheoid anomuran, and lobster species standardised per 20 Data primarily based on Wienberg Rasmussen *et al*. [18]; Schweitzer & Feldmann [75], [124], [125]; Schweitzer *et al*. [126]; Ceccon & De Angeli [20]; Robins *et al*. [19]; and herein. See Table 3 for additional references. Swellings have not been found in non-galatheoid anomurans and only one infested shrimp is known [182].

**Figure 6:**
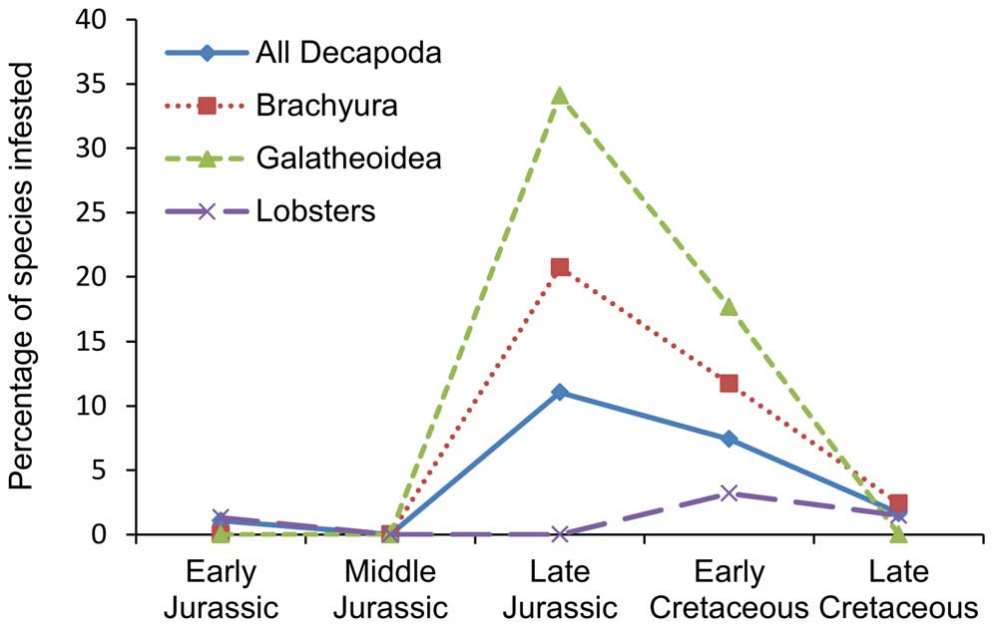
The percentage of marine decapod, brachyuran, galatheoid anomuran, and lobster species infested per Jurassic and Cretaceous epoch.

Plotting the infestation percentage against number of specimens for taxa with at least 30 specimens yields a positive, significant correlation on the species and genus level, but not on the family level (Fig. 4). The number of species and the percentages of species affected per epoch show that infestation is highest in the Late Jurassic for all Decapoda, Brachyura, and galatheoid anomurans, whereas infestation in lobsters is too low to make general statements (Figs. 5, 6; Tables 2, 3). More specifically, 11% of all decapod species are infested in the Late Jurassic, mainly caused by high percentages of infestation in Late Jurassic galatheoid anomurans (34%) and Brachyura (21%), whereas these percentages are substantially lower in the epochs prior to and subsequent of the Late Jurassic (Fig. 6). Non-galatheoid anomurans with these swellings are unknown from the fossil record, whereas only one fossil shrimp species shows such a swelling [182]. In total, 88 infested species are now known from the fossil record (Tables 2, 3), which is more than a 50% increase compared to the list in Wienberg Rasmussen *et al*. [18]. The decapod species containing such a swelling include 25 anomurans, 55 brachyurans, seven lobsters, and one shrimp (Tables 2, 3).

**Table 2:** The number of infested marine decapod, brachyuran, galatheoid anomuran, lobster, and shrimp species per post-Triassic epoch.

Epoch	All Decapoda	Brachyura	Galatheoid anomurans	Lobsters	Shrimps
Holocene (excl. today)					
Pleistocene	1	1			
Pliocene	1	1			
Miocene	6	3	3		
Oligocene					
Eocene	8	4	3	1	
Paleocene	3	2	1		
Late Cretaceous	8	6		2	
Early Cretaceous	22	15	3	3	1
Late Jurassic	38	23	15		
Middle Jurassic					
Early Jurassic	1			1	
**Total**	**88**	**55**	**25**	**7**	**1**

Data primarily based on Wienberg Rasmussen *et al*. [18]; Schweitzer & Feldmann [75], [124], [125]; Schweitzer *et al*. [126]; Ceccon & De Angeli [20]; Robins *et al*. [19]; and herein. See Table 3 for additional references. Swellings have not been found in non-galatheoid anomurans.

**Table 3:** All known fossil decapod crustacean species exhibiting a swelling in at least one specimen.

Anomuran (A),brachyuran (B),lobster (L) orshrimp (S) family	Genus and species	Period andEpoch	Stage	Countryof origin	Sourceused fordataherein
(A) Munidopsidae	*Ambulocapsa sentosa* [19]	Late Jurassic	Tithonian	Austria	[19]
(A) Munidopsidae	*Bullariscus triquetrus* [19]	Late Jurassic	Tithonian	Austria	[19]
(A) Munidopsidae	*Cracensigillatus acutirostrus* ([134])	Late Jurassic	Tithonian	Austria	[19]
(A) Munidopsidae	*C. gracilirostrus* [19]	Late Jurassic	Tithonian	Austria	[19]
(A) Munidopsidae	*C. prolatus* [19]	Late Jurassic	Tithonian	Austria	[19]
(A) Munidopsidae	*Culmenformosa glaessneri* [19]	Late Jurassic	Tithonian	Austria	[19]
(A) Munidopsidae	*Gastrosacus eminens* ([135])	Late Jurassic	Tithonian	Austria	[19]
(A) Munidopsidae	*G.? latirostrus* ([136])	Late Jurassic	Oxfordian	France	[18]
(A) Munidopsidae	*G. meyeri* ([134])	Late Jurassic	Tithonian	Austria	[18]
(A) Munidopsidae	*G. tuberosiformus* ([137])	Late Jurassic	Tithonian	Austria	[19]
(A) Munidopsidae	*G. wetzleri* [138]	Late Jurassic	Kimmeridgian,Tithonian	Czech Republic,France, Germany,	[18]
(A) Galatheidae	*Eomunidopsis aldoirarensis* [32]	Early Cretaceous	Albian	Spain	[32], herein
(A) Galatheidae	*E. navarrensis* ([127])	Early Cretaceous	Albian	Spain	[32], herein
(A) Galatheidae	*E. neojurensis* [139] ( = *Galathea antiqua* [134])	Late Jurassic	Tithonian	Austria,Czech Republic	[15], [18]
(A) Galatheidae	*Galathea weinfurteri* [140]	Neogene, Miocene	Serravallian*	Hungary	[18]
(A) Galatheidae	*Galatheites zitteli* ([134])	Late Jurassic	Tithonian	Austria,Czech Republic	[18]
(A) Galatheidae	*Lessinigalathea regale* [141]	Paleogene, Eocene	Ypresian	Italy	[20]
(A) Galatheidae	*Mesogalathea striata* ([142])	Late Jurassic	Tithonian	Austria,Czech Republic	[18]
(A) Galatheidae	*Palaeomunida multicristata* [141]	Paleogene, Eocene	Priabonian	Italy	[20]
(A) Galatheidae	*Paragalathea ruizi* ([127])	Early Cretaceous	Albian	Spain	herein
(A) Galatheidae	*P. verrucosa* ([134])	Late Jurassic	Tithonian	Czech Republic	[18]
(A) Munididae	*Protomunida munidoides* ([143])	Paleogene, Paleocene	Danian	Denmark	[18]
(A) Porcellanidae	*Lobipetrolistes blowi* [141]	Paleogene, Eocene	Priabonian	Italy	[20]
(A) Porcellanidae	*Petrolisthes magnus* [144]	Neogene, Miocene	Serravallian*	Hungary	[18]
(A) Porcellanidae	*Pisidia* cf. *P. kokayi* ([144])	Neogene, Miocene	Langhian*	Hungary	[18]
(B) Dromiidae	*Kromtitis* cf. *K. koberiformis* [145]	Paleogene, Eocene	Priabonian	Italy	[20]
(B) Dynomenidae	*Cyamocarcinus angustifrons* [146]	Paleogene, Eocene	Ypresian	Italy	[20]
(B) Dynomenidae	*Cyclothyreus divaricatus* [75]	Late Jurassic	Tithonian	Austria	[75]
(B) Dynomenidae	*C. latus* ([134])	Late Jurassic	Tithonian	Czech Republic	[18]
(B) Dynomenidae	*C. quadrophthalmus* [75]	Late Jurassic	Tithonian	Austria	[75]
(B) Dynomenidae	*C. reussi* ([147])	Late Jurassic	Tithonian	Austria	[17], [18]
(B) Dynomenidae	*C. strambergensis* [142]	Late Jurassic	Tithonian	Czech Republic	[17], [18]
(B) Dynomenidae	*C. tithonius* ([147])	Late Jurassic	Tithonian	Italy	[17], [18]
(B) Dynomenidae	*Graptocarcinus texanus* [130]	Early Cretaceous	Albian	Spain	herein
(B) Longodromitidae	*Abyssophthalmus mirus* ([134])	Late Jurassic	Kimmeridgian*	Germany	[18], [72]
(B) Longodromitidae	*A. spinosus* ([24])	Late Jurassic	Oxfordian*	Germany	[17], [18]
(B) Longodromitidae	*Longodromites excisus* ([26])	Late Jurassic	Oxfordian	Germany	[17], [18]
(B) Longodromitidae	*L. ovalis* ([134])	Late Jurassic	Tithonian	Czech Republic	[17], [18]
(B) Longodromitidae	*Planoprosopon heydeni* ([26])	Late Jurassic	Oxfordian	Poland	[18]
(B) Nodoprosopidae	*Nodoprosopon? katholickyi* ([142])	Late Jurassic	Tithonian	Czech Republic	[18]
(B) Nodoprosopidae	*N. ornatum* ([148])	Late Jurassic	Kimmeridgian*	Germany	[18], [72]
(B) Goniodromitidae	*Cycloprosopon complanatiforme* ([134])	Late Jurassic	Tithonian	Czech Republic	[18]
(B) Goniodromitidae	*C. octonarium* [125]	Late Jurassic	Tithonian	Austria	[125]
(B) Goniodromitidae	*Distefania renefraaijei* [34]	Early Cretaceous	Albian	Spain	[34], herein
(B) Goniodromitidae	*D. oxythyreiformis* ([147])	Late Jurassic	Tithonian	Czech Republic	[18]
(B) Goniodromitidae	*Eodromites grandis* ([26])	Late Jurassic	–	Poland	[124], [125]
(B) Goniodromitidae	*E. rostratus* ([25])	Late Jurassic	Oxfordian	Poland	[18]
(B) Goniodromitidae	*Goniodromites bidentatus* [149]	Late Jurassic	Tithonian	Czech Republic	[18]
(B) Goniodromitidae	*G. globosa* ([142])	Late Jurassic	Tithonian	Czech Republic	[18]
(B) Goniodromitidae	*G. laevis* ([127])	Early Cretaceous	Albian	Spain	herein
(B) Goniodromitidae	*G. polyodon* [149] ( = *G. complanatus* [149])	Late Jurassic	Tithonian	Czech Republic	[17], [18]
(B) Goniodromitidae	*Palaeodromites octodentatus* [150]	Early Cretaceous	Hauterivian	France	[18]
(B) Goniodromitidae	*Pithonoton marginatum* [24]	Late Jurassic	Oxfordian,Tithonian	Czech Republic,Poland	[18]
(B) Goniodromitidae	*Sabellidromites scarabaea* ([151])	Early Cretaceous	Albian	England	[124]
(B) Prosopidae	*Acareprosopon bouvieri* ([133])	Early Cretaceous	Albian	Spain	herein
(B) Prosopidae	*Prosopon aculeatum* [26]	Late Jurassic	?Tithonian*	Germany	[18]
(B) Homolidae	*Latheticocarcinus atlanticus* ([152])	Late Cretaceous	Campanian	USA	[18]
(B) Torynommidae	*Torynomma flemingi* [153]	Late Cretaceous	Maastrichtian*	New Zealand	[18]
(B) Torynommidae	*Withersella crepitans* [131]	Early Cretaceous	Aptian	England	[18]
(B) Homolodromiidae	*Notiodromia australis* ([154])	Late Cretaceous	Campanian*	Antarctica	[18]
(B) ?Macropipidae	*Faksecarcinus* (cf.) *koskobiloensis* ([28])	Early Cretaceous	Albian	Spain	herein
(B) Palaeocorystidae	*Cretacoranina testacea* ([155])	Late Cretaceous	Maastrichtian*	USA	[18]
(B) Palaeocorystidae	*Eucorystes carteri* ([156])	Early Cretaceous	Albian	England	[18]
(B) Palaeocorystidae	*Joeranina platys* ([157])	Late Cretaceous	Santonian	Canada	[126]
(B) Palaeocorystidae	*Notopocorystes stokesii* ([158])	Early Cretaceous	Albian	England, France	[18], [48], [159]
(B) Palaeocorystidae	*N. serotinus* [131]	Early Cretaceous	Albian	England	[18]
(B) Lyreididae	*Macroacaena rosenkrantzi* ([27])	Late Cretaceous	Maastrichtian	Greenland	[18]
(B) Raninidae	*Lophoranina marestiana* ([160])	Paleogene, Eocene	“middle”	Italy	[20]
(B) Raninidae	*Notosceles bournei* [161]	Paleogene, Paleocene	Danian*	USA	[18]
(B) Raninidae	*Quasilaeviranina ovalis* ([162])	Paleogene, Paleocene	Danian*	USA	[18]
(B) Feldmanniidae	*Caloxanthus paraornatus* [28]	Early Cretaceous	Albian	Spain	herein
(B) Feldmanniidae	*Feldmannia wintoni* ([162])	Early Cretaceous	Albian*	USA	[18]
(B) Aethridae	*Hepatus lineatinus* [163]	Neogene, Miocene	Serravallian-Tortonian*	Panama	[18]
(B) Necrocarcinidae	*Necrocarcinus labeschii* ([164])	Early Cretaceous	Albian	England	[18]
(B) Pilumnidae	*Galenopsis similis* [165]	Paleogene, Eocene	Ypresian	Italy	[20]
(B) Portunidae	*Charybdis* sp. [166]	Neogene, Pliocene	?Zanclean*	Japan	[18]
(B) Portunidae	*Portunus woodwardi* [167]	Neogene, Miocene	–	Brunei	[18]
(B) Leucosiidae	*Philyra granulosa* [167]	Neogene, Miocene	–	Brunei	[18]
(B) Leucosiidae	*P. syndactyla* [119]	Quaternary, Pleistocene	Middle*	Japan	[18]
(B) Viaiidae	*Viaia robusta* [35]	Early Cretaceous	Albian	Spain	[35], herein
(L) Mecochiridae	*Mecochirus rostratus* [27]	Late Cretaceous	Maastrichtian	Greenland	[18]
(L) Nephropidae	*Hoploparia dentata* ([168])	Early Cretaceous	Hauterivian	England	[18]
(L) Nephropidae	*H. gammaroides* [169]	Paleogene, Eocene	Ypresian*	England	[18]
(L) Nephropidae	*Hoploparia trigeri* ([170])	Late Cretaceous	Cenomanian	France	[18]
(L) Erymidae	*Eryma* sp. [118]	Early Jurassic	Toarcian	Indonesia	[18]
(L) Erymidae	*Palaeastacus? scaber* ([132])	Early Cretaceous	Albian	England	[18]
(L) Erymidae	*Pustulina? granulata* ([132])	Early Cretaceous	Albian	England	[18]
(S) Axiidae	*Axiopsis sampsonumae* [182]	Early Cretaceous	Albian	USA	[182]

* = corrected or added compared to Wienberg Rasmussen *et al.* [18] for those with that source.

## Discussion

### Quantitative Data Koskobilo Fauna

Considerable variation in infestation percentages can be found within the Galatheidae, but also within the genus *Eomunidopsis*, supporting the need to determine infestation percentages at the species level to infer possible causes of this variation in the fossil record. Variable infestation percentages for congeners were also noted by Wienberg Rasmussen *et al*. [18] for *Macroacaena rosenkrantzi* and the contemporary *M. succedana* from the Maastrichtian of Greenland (as noted above) and by Rayner [40] for modern *Munida gregaria* ([41]) (3.7% infested) and *M. subrugosa* [42] (7.4%), although *M. subrugosa* currently is considered a synonym of *M. gregaria* (e.g., [43]).

The fact that galatheids are more affected than any other family in this case could suggest that they were more prone to infestation than other taxa. Another hypothesis is that the percentage of infestation is positively influenced by the number of specimens: more specimens yield a higher percentage of infestation (Fig. 4) because of specialisation of isopods on abundant decapod taxa. This could increase their chances of finding a suitable host. However, it should be noted that data on the host-specificity of modern isopods on decapods are sparse [8], [44], [45], and that the trend is mostly driven by a high infestation percentage in a single species (*Eomunidopsis navarrensis*), suggesting that more research is needed to confirm or reject this hypothesis.

Life mode may also affect infestation percentages. For example, Rayner [40] noted that trawled (presumably mostly epifaunal) specimens of *Munida gregaria* were infested in 3.7% of cases, whereas only 2.9 and 1.9% of specimens in two samples of juvenile, pelagic conspecifics were infested. However, almost all extant galatheoids are benthic in their post-larval stage (e.g., [46]), which suggests that variation in infestation percentage within this family may not be explained by variation in this type of life habit. Most brachyurans from the Koskobilo fauna probably were benthic as well, with the possible exception of *Faksecarcinus* (cf.) *koskobiloensis* given its wide, short (heightwise) carapace shape. It may also be hypothesised that semi-epifaunal or infaunal decapods could be less likely to be exposed to water containing the isopod larvae seeking a decapod host. Hence, epifaunal and swimming decapods could have higher percentages of infestation than semi-epifaunal or infaunal decapods. Carapaces of the latter such as raninoids, certain lobsters, and thalassinids (e.g., [47]–[49]) are usually longer than wide to promote burrowing. The hypothesis is not supported by decapods from the Koskobilo fauna as well-represented genera within Goniodromitidae (*Distefania, Eodromites,* and *Goniodromites*) and *Graptocarcinus* are wider than long or about equally wide as long [29], [34], and, thus, may have been epifaunal, yet are hardly infested. Moreover, modern thalassinids are known to be infested [50]. Differences in life mode are, thus, not useful to explain the differences in infestation percentages when comparing Brachyura to Anomura here.

Another, hypothetical explanation may lie in the intrinsic effectiveness of keeping the isopod larvae out of the gill chambers and the effectiveness of removing these isopods once infested. Bauer [51] indicated that water in modern decapods is pumped into the gill chambers via a process of the maxilla, the scaphognathite, and that limiting the entry of material into the gills as well as cleaning is necessary because of the possibility of fouling by particulate matter, the growth of microbes, and epizoites. To this end, the water flow can be reversed, the incurrent opening of many decapods is protected by filters of setae, and decapods have several cleaning mechanisms often involving brushing by setae located on specialised parts of the extremities [51], [52]. It must be concluded that the cleaning and preventing mechanisms failed to avoid and remove the entry and development of parasitic isopods in case of a visible infestation. In addition to the above-mentioned reasons, the differences in infestation percentages on the species, genus, and family level may be attributed to the relative effectiveness of these mechanisms, assuming they already had developed by mid-Cretaceous times. The higher-than-average infestation in galatheoids may be related to a relatively low effectiveness of the gill cleaning mechanism in galatheoids compared to Brachyura. Bauer [52] noted that crabs typically make use of setiferous epipods on the maxillipeds to clean the gills instead of the specialised chelate fifth pereiopod pair in the galatheoid *Pleuroncodes planipes* [53]. However, the relative effectiveness of these two mechanisms with respect to avoiding parasitism by isopods is unknown to us and deserves more research.

### Potential Biases in the Koskobilo Fauna

The percentages of infestation provided for the Koskobilo fauna and for other faunas may have been influenced by several biases. Williams & Boyko [8] noted that some bopyrid isopods of the subfamily Athelginae are parasitic on the abdomina of hermit and king crabs (Anomura), whereas the Hemiarthrinae and Phyllodurinae are parasitic on the abdomina of shrimps, although no true abdominal swellings are observed. In the present paper, possible swellings on the abdomina could not be observed as complete anomuran abdomina have not been found yet and shrimps are unknown from the fauna.

The percentages for the entire fauna and per species may represent maxima from the perspective that not all swellings in modern, living, infested decapods contain parasitic isopods anymore, albeit uncommon [12]. These parasites may have been dislodged during the moulting phase [54].

Another bias could be the preferential breakage of carapaces with (a) swelling(s), thereby artificially lowering the true percentage of affected taxa and specimens. For example, McDermott [11] noted that the infested branchial chamber of a specimen of the extant brachyuran *Pachygrapsus transversus* ([55]) was damaged. Actualistic experiments should substantiate or refute such an assumption, also by considering possible differences in cuticle thickness both over and away from the swelling, which is hardly investigated (but see Bursey [56], who found that the inner part of the cuticle was thicker in a single species of galatheoid).

The percentage of the entire fauna could have been influenced by a relatively high or low preservation potential of taxa with swelling percentages that deviate from the average. These include, for example, paguroid anomurans and caridean shrimps, which are heavily infested in modern environments (e.g., [50]), but (useful) specimens are not found in Koskobilo. A deviant from average preservation potential of specimens of the highly infestated and abundant species *Eomunidopsis navarrensis* (see Table 1) may have influenced the overall infestation percentage.

Isopods are not expressed as a swelling on the carapace when they are too small to affect the carapace (see McDermott [11] for a modern example), thereby lowering the actual percentage of affected decapod specimens of a fauna. This may especially be the case for species with a large maximum size for which the isopod has to obtain a substantial size to be visible as a swelling expressed on the branchial region. Indeed, Roccatagliata & Lovrich [12] noted that the growth rate of the isopod parasite started to lag behind that of the extant anomuran *Paralomis granulosa* ([57]) once this species reached a carapace length of 30 mm. Thus, generally lower observable infestation percentages may be hypothesised in the fossil record for species with a larger maximum size compared to species with small maximum size. Although common species (at least 30 specimens present) with a relatively large maximum size from the Koskobilo quarry show low infestation percentages (*Distefania incerta, Goniodromites laevis,* and *Graptocarcinus texanus*) (Table 1), so do part of the small species (*Acareprosopon bouvieri, Caloxanthus paraornatus,* and *Paragalathea ruizi*), thereby only in part supporting the hypothesis. On the other hand, decapods in this fauna are generally of small size (see Klompmaker [29] and references therein).

Another factor that could have suppressed the overall and per taxon infestation percentages here is the fact that part of the extant decapods have a final moulting phase, either when they reach maturity or during maturity (e.g., [58], [59]). The majority of brachyurans appear to have determinate growth [58], [59], whereas not much is known of the other frequently infested group, the galatheoids, except for *Cervimunida johni* [60], in which growth appears indeterminate [61]. When an isopod parasite infests the decapod after the final moulting phase or is not large enough to bulge the cuticle, then a swelling is unlikely to become visible on the branchial chamber of the carapace, although it should be noted that infestation by isopods was typically found in young individuals of the extant anomuran *P. granulosa* [12]. If decapods already experienced a final moulting phase in the late Albian, then the apparent infestation percentages are lower than in actuality, and, therefore, the percentages must be considered minimum estimates with respect to decapod size and the final moult. On the other hand, larger specimens may be able to get rid of parasitic isopods or outlive them as no isopod was found underneath the branchial swelling for some extant decapods [12], [54], [62]. If the swelling is not retained during the following moult phase, then no evidence exists of past infestation and the effect of potentially artificially increasing the infestation percentage would be limited.

A complicating factor in the fossil record is the mixture of moults and corpses in the sample, which cannot be distinguished in the case of Koskobilo [37]. Given that the growth rate of decapods can decrease due to infestation (e.g., [62], [63]), fewer moults will likely be produced per time unit compared to uninfested, conspecific specimens. This may result in an underestimation of the true percentage of infestation on the taxon and ultimately on the assemblage level.

### Infestation through Time

Wienberg Rasmussen *et al*. [18] showed the number of affected species per family (which is, in part, a function of the number of taxa within a family) and they also indicated (p. 35) that the highest number of decapod species (not crab, contrary to their claim) infested originated from the Jurassic. Fewer affected species are known from the Cenozoic. The comparative paucity of swellings of Cenozoic age was already noted by Houša [15], and this observation has been substantiated by Robins *et al*. [19] and results presented herein, yet not by Ceccon & De Angeli [20]. This pattern is supported by quantitative data (Tables 2, 3) showing high infestation percentages in the Late Jurassic, even when corrected for the unequal duration of epochs (Fig. 5) and as percentages per epoch (Fig. 6). It is known from modern oceans that the distribution patterns of epicarideans are influenced by their intermediate (copepod) and their final hosts (always crustaceans; see [8]). It is likely that their distribution and diversity through time is influenced by their hosts as well. Several hypotheses can be put forward as to why there may be an apparent lower number of infested species and lower percentages of infestion in the Cenozoic and latest part of the Mesozoic:

#### Adaptations increasing resistance

Conway Morris [3] speculated that the post-Jurassic decline could have been caused by the evolution of adaptations with the goal of resisting infestation in decapod crustaceans, but he also indicated that rigorous testing was called for. An important question is whether or not becoming resistant to infestation by isopods happened, and, if so, was controlled by evolutionary innovation. This may not have been the case, given the generally low incidence of infestation known from the fossil record (see introduction, Fig. 5) and the non-lethal consequences of infestation. As mentioned above, the intrinsic effectiveness of keeping the isopod larvae (and other fouling organisms) out of the gill chambers and the effectiveness of removing the isopods once infested may play a role in explaining variations in percentages of infestation in the Koskobilo fauna. This effectiveness may have changed through time as a function of changing decapod faunas that were able to keep fouling organisms out of their gill chambers more effectively. For example, Jurassic and Early Cretaceous brachyurans almost exclusively consist of primitive crabs (Podotremata), whereas more advanced crabs (Eubrachyura) became progressively more abundant from the mid-Cretaceous onwards. A possible indication of the validity of the hypothesis that eubrachyurans are more effective in keeping isopod larvae out is to study infestation patterns in modern Podotremata and Eubrachyura from the same general area and habitat, and with a similar lifestyle.

#### Diversity changes of commonly infested groups

During the Late Jurassic and mid-Cretaceous, galatheoids were common in reef settings, both in terms of percentages of species amongst all decapods (Table 4) and in terms of specimens ([29], [64], 483/1,078 = 45%). Galatheoid abundance and species richness in reefs appear to have decreased since that time (Table 4), unrelated to reef abundance (see [65], [66]). Swellings are common in reef-associated Jurassic and mid-Cretaceous galatheoids (see [18], [19], herein). Their declining contribution to reef faunas, from which most extinct galatheoids have been described, may in part explain why fewer swellings are found in the Cenozoic. Furthermore, Wienberg Rasmussen *et al*. [18] also listed a number of Tithonian goniodromitid crabs with swellings (their table 2). Even though goniodromitids are common in the Koskobilo fauna, only two specimens with a swelling have been noted to date (Table 1). This family had its acme in the Late Jurassic (when also several species were infested), experienced a possible re-diversification during the mid-Cretaceous, and disappeared during the Paleocene [34], which may have negatively influenced isopod infestation. The same could be suggested for Palaeocorystidae, a raninoid family that was most speciose in the mid-Cretaceous, but declined in species diversity towards the end of the Cretaceous; to date, no members of this family are known from the Cenozoic (see [48]). Wienberg Rasmussen *et al*. [18] showed that most infested species of this family are found in the mid-Cretaceous. The hypothesis that changing diversity of commonly infested groups could have affected infestation percentages through time is supported by the fact that a part of extant bopyrids seem host-specific as they infest a single decapod species [44], [45], leaving them vulnerable when the decapod host goes extinct.

**Table 4:** Decapod and galatheoid anomuran species richness and percentage of galatheoids in Mesozoic and Cenozoic reef-associated faunas.

Localities	Formation	Age rock unit	Total decapodspecies	Galatheoidspecies	Percentagegalatheoids	Referencesused
Ernstbrunn quarries,Austria	ErnstbrunnLimestone	Upper Jurassic(Tithonian)	83	35	42.2	[19, 73–76, 124,125, 171–177]
Koskobilo quarry,Spain	Eguino	mid-Cretaceous(Albian)	36	10	27.8	[29]
ENCI quarry,The Netherlands	Maastricht	Upper Cretaceous(Maastrichtian)	30	2	6.7	[29], [178]
Faxe quarry,Denmark	Faxe	Lower Paleocene(Danian)	20	4	20.0	[179]
exposures of SzépvölgyLimestone Formation, Hungary	SzépvölgyLimestone	Upper Eocene(Priabonian)	58	9	15.5	[180]
sites in the Börzsöny- andPilis Mountains, Hungary	–	mid-Miocene(Langhian)	19	1	5.3	[144]
localities near Budapestand Diósd, Hungary	–	mid-Miocene(Serravallian)	15	1	6.7	[144]
Depiru Beds, Malta	Upper CorallineLimestone	Upper Miocene(Messinian)	19	3	15.8	[181]

#### Copepods

Pelagic calanoid copepods are the intermediate hosts of all known epicaridean isopods in the modern marine realm [8]. Unfortunately, only a handful of fossil copepods are known because of their small size and fragility [67]; these do not include calanoids. A hypothesis for the drop in infested species during the Cenozoic and latest part of the Cretaceous, in comparison to the Late Jurassic, may be a move of calanoid copepods to deeper waters. The larvae, following the intermediate copepod-phase (see [8]), could then also be found mostly in waters where sediments have a lower preservation potential than strata laid down at shallower depths. Bradford-Grieve [68] suggested that the related clausocalanoid copepods may have found refuge in benthic habitats in deeper waters after the mid-Cretaceous oxygen minimum, but that they could have reinvaded the entire water column during the Cenozoic. Alternatively, calanoid copepods may have become less abundant in Late Cretaceous, Paleogene and Neogene oceans. More fossil copepods need to be found in order to confirm or reject these speculative hypotheses.

#### Changes in decapod size

Preliminary data and personal observations suggest that the maximum size of decapods increased throughout geological time with Cenozoic and Late Cretaceous decapods being larger than their (earlier) Mesozoic counterparts [69]. If the median size of decapods also increased, then isopod parasites would be more difficult to observe in fossil decapod assemblages because it would take more time for the isopods to reach a size that would affect the cuticle of the branchial region for those decapods infested during adulthood. This would suppress the infestation percentages in fossil decapods. However, as noted above, infestation may primarily occur in small specimens of a species [12], [62], [70]. Along with a larger decapod size, decapod cuticle also becomes thicker as cuticle thickness increases with ontogeny (e.g., [71]) and may become possibly harder to deform (see [11]) during the intermoult periods when decapods may still grow slightly [58]. Additionally, it may also become more difficult to deform the developing cuticle during the moult phase. Such swellings in fossil decapods are usually smaller than ∼10 mm (e.g., [18], herein). The largest swelling known to us is ∼16 mm in length in the brachyuran *Lophoranina marestiana* ([160]) with a carapace length of 35 mm as figured in Ceccon & De Angeli [20] (their pl. 2.1).

#### Collecting bias

Of note is that the majority of Jurassic species of infested decapod crustaceans is known from reef settings, in which relatively numerous decapod species (and specimens) can be found (e.g., [19], [72]–[76]) in comparison to other environments (Recent, [77]; Cretaceous, [29]). Additionally, workers have been collecting in some these strata for a long time, which should have increased the likelihood of finding infested decapods with many species and specimens available. This may, at least in part, explain why many infested species are known from the Jurassic and why so many infested decapod species are reported from the Koskobilo quarry (33%). On the other hand, decapod diversity is known to have been anomalously high in the Late Jurassic compared to other time intervals during the Mesozoic [39], [78], [79], which is a direct function of the ubiquitous presence of reefs [39], [79]. Thus, numerous species with swellings could be expected from the Late Jurassic. However, it should be noted that the coral-associated sediments of the type Maastrichtian in the Netherlands are also highly diverse, but not a single specimen with a swelling has been found after intensive collecting. Also, only one infested specimen is figured from the coral-associated, diverse decapod assemblage from the Paleocene (Danian) of Faxe in Denmark [18], [179] (Table 4). Indeed, specimens with such swellings are extremely rare from these deposits (Sten Jakobsen, pers. comm. to AAK, February 2014). These results support the validity of the observed trend of lower infestation percentages since the Late Jurassic.

#### Reporting bias

Cenozoic swellings may simply have been under-recorded; however, they do appear to be uncommon primarily during this interval (RHBF, pers. obs.; Roger Portell, pers. comm. to AAK, February 2013).

### Other Deformities on Decapods caused by Parasites

Another example of parasitic behaviour that affects the morphology of decapod crustaceans in the fossil record is the castration of the brachyuran *Tumidocarcinus giganteus* [80] from the Miocene of New Zealand. Feldmann [81] attributed this to rhizocephalan barnacles. Earlier, Bishop [82], [83] had described sexually aberrant specimens of the Late Cretaceous brachyuran *Dakoticancer overanus* [84] from South Dakota (USA), which may or may not have been caused by parasitism [81]. Jones [85] suggested parasitism as the most likely cause for the existence of intersex specimens of *D. overanus*. Other parasite-induced deformities have been documented from extant crabs. For example, Miyashita [86] documented specimens of the extant brachyuran *Eriocheir japonica* ([87]) with slightly asymmetrical carapaces caused by an entoniscid isopod, while Shield & Kuris [88] noted that several regions of modern-day brachyurans (*Hemigrapsus* spp.) can be affected by entoniscid isopods as well. Similar examples may be expected from the fossil record.

## Systematic Ichnology

### Remarks

Bromley [89] was the first to make a formal distinction between borings in hard substrates, embedment cavities, burrows in soft substrates and other forms of destruction of hard substrates by bioerosion. He noted (p. 51) that, ‘The lodgment of a non-boring parasite in the calcareous skeleton of a host organism can cause great deformity of the skeletal material. The resulting cavity can be distinguished easily from a boring. Such cavities have been described by Voigt [90], [91] in Cretaceous octocorals as the work of parasitic ascothoracican cirripeds.’ Further on in the same paper (p. 67) it reads, ‘As parasites, some ascothoracicans produce cavities in organic skeletons by causing deformation of the growth of the host. Voigt [90], [91] has attributed deformity of this kind in Cretaceous octocorals to ascothoracican cirripeds but the cavities must be considered a result of embedment rather than of boring.’ Palmer & Wilson [92] coined the term ‘bioclaustration’ for the process by which a living skeletal-secreting organism overgrows a living counterpart. In a recent overview of the stratigraphy of marine bioerosion, this theme is covered anew [93], ‘Voigt [90], [91] attributed cyst-like cavities, having a slit-like entrance, in Danian octocorals to the parasitism of ascothoracicans, naming the (unpreserved) animals *Endosacculus moltkiae* [90] and *Endosacculus*? *najdini* [91]. These may be bioclaustration structures.’ Such gall-like ‘cysts’ generally have a drop- or keyhole-shaped entrance (see [90], [94]), which is lacking from the swellings described herein. At some point in time, larvae of isopods must have entered the anomuran, brachyuran, and lobster carapaces they infested (see f.e. [8]) through the branchial openings to attach to the gill filaments. This is not expected to have left any (bioerosional) signature on the decapod crustacean host; in short, only the swelling testifies to the former presence of such a settler symbiont (*sensu* [95]) of a bioclaustration (embedment) signature. Tapanila & Ekdale [95] recently clarified the ethological nomenclature of such structures, in noting that, ‘Extension of the bioclaustration cavity requires not only that the host skeleton continues to grow but also that the settler symbiont remains alive. … The resulting trace fossil therefore preserves dual activities on the part of the embedded settler: interference of host growth, and maintenance of a dwelling structure. Trace fossils produced in this way belong to the ethologic category, impedichnia [96].’ Such isopod infestation is clearly an example of parasitism as it negatively affects the infested decapods: a lower fecundity or even castration may occur, secondary sex characters can be modified, distortion of the epipodites has been reported, and growth rate can be negatively affected (e.g., [8], [11], [54], [62], [70], [97], [98]). The ethologic category impedichnia could be used to describe this particular parasite-host interaction.

Recently, Bertling *et al*. [99] cast doubt on whether bioclaustration (embedment) structures should be called trace fossils as they referred to them as in the ‘grey zone’ and ‘non-traces’. Later on, Bertling *et al*. [99], however, also mentioned that ‘for convenience and in order to maintain stability of names with a high ecological meaning, we advocate nomenclaturally (not taxonomically) treating them as if they were ichnotaxa’ in referring to at least hybrid structures including bioclaustrations in which boring and overgrowth both are evident. Whether bioclaustrations should or should not be called trace fossils seems still under debate as several authors do refer to them as trace fossils (e.g., [95], [100]–[104]).

Whether eventually considered true trace fossils or not, we here recognise that several criteria used by Bertling *et al*. [99] to consider bioclaustration structures as non-traces do not apply to the swellings described herein. Skeletal parts are moved in this case due to the presence of a growing isopod parasite pushing against the cuticle (see [11], Christopher Boyko, pers. comm. to AAK, December 2013), which is probably a reason for stretching and/or obscuration of ornamentation in examples herein (Figs. 1A, 2E, 3A, 3M) and as shown in the literature on fossil decapods (e.g., [18]: pls 3.1, 3.3; [19]: fig. 16.12; [20]: pl. 1.1). The substrate is actively manipulated by the infesting isopod as Bursey [56] showed that the mandibles penetrated or bored into the inner decapod cuticle, probably the reason for a thickening of the cuticle locally. Bioimmuration (defined here as the overgrowing of dead organisms, as in Bertling *et al*. [99]) is generally not applicable in our case as a living isopod larva needs to get into the branchial chamber of a decapod first after which the juvenile epicaridean grows before such a swelling will develop, although the parasite may die within the branchial chamber. Such swellings typically are evidence for the presence of a living isopod parasite. Lastly, these swellings are very distinct and not a growth variation of unclear origin. As suggested by Bertling *et al.* [99], we treat these swellings (hybrid structures) at least nomenclaturally as ichnotaxa by erecting a new ichnogenus and -species to cover all of such traces.

Ichnogenus *Kanthyloma* nov. urn:lsid:zoobank.org:act:684FA82A-9B4B-45C3-92E5-814C3874F203.

#### Etymology

Derived from the Greek *kanthyle* meaning swelling or tumour. The gender is feminine.

#### Type ichnospecies

*Kanthyloma crusta* isp. nov.

#### Diagnosis

Subcircular to suboval swelling on a convex surface around a structure of biogenic origin not associated with any perforations, holes, or other irregularities.

#### Remarks

The works of Pickerill [105], Bertling [106], and Tapanila & Ekdale [95] were followed in identifying valid ichnotaxabases by focusing on the morphology of the structure primarily. Other examples of bioclaustration expressed as swellings are known from the fossil record, but they differ from the new ichnogenus and have often not been described using Linnaean nomenclature. Two copepod-induced swellings in echinoids, referred to as the ichnotaxon *Castexia douvillei* [107] and the so-called ‘Halloween pumpkin-mask’ cysts, both of which are suggested to be treatable as trace fossils by Radwańska & Radwański [108], are different in that both bear numerous perforations in the swelling (e.g., [108]: figs. 5–7, [109]: figs. 4, 5). Halloween pumpkin-mask cysts are also known from fossil crinoid and modern hydrocoral stems ([108]: figs. 5.4, 8). Abdelhamid [110] reported on swellings on tests of Cretaceous echinoids from Egypt, but these swellings were associated with borings and were not named. A fossil echinoid spine bears an inferred copepod-induced swelling, but this swelling is irregular and no ichnotaxon was erected ([108]: fig. 9e). Fossil crinoid stems and arms have also been shown to contain swellings (e.g., [111]–[113]), but these swellings, linked to myzostomid annelid parasites, exhibit holes typically. The ichnofossil *Myzostomitus clarkei* [114] ( = *Tremichnus cysticus* [115] *sensu* [108]) has been used for swellings containing pits in crinoid stems (see [115]: fig. 3). Roman [116] described similar swellings from Miocene echinoids. Swellings associated with elliptical and circular concavities caused by coral attachment to Devonian crinoid stems were discussed and described ichnotaxonomically [117].

*Kanthyloma crusta* isp. nov. (Figs. 2, 3) urn:lsid:zoobank.org:act:FA05E57B-57B0-4BF1-A6BF-17A8F285A528.

#### Etymology

Derived from the Latin *crusta* which translates as the hard outer shell.

#### Type material and type locality

The holotype (MGSB78340) is found in *Eomunidopsis navarrensis* (MGSB78339, Fig. 2C, D); paratypes (MAB k. 2601-i, 3008-i and 3177-i) occur in *Distefania renefraaijei* (MAB k. 2601, Fig. 3E, F), *Paragalathea ruizi* (MAB k. 3008, Fig. 2A, B), and *Caloxanthus paraornatus* (MAB k. 3177, Fig. 3G–I), respectively. All material originates from mid-Cretaceous (upper Lower Cretaceous, upper Albian) limestones of the Eguino Formation (Albeniz Unit) in northern Spain [29].

#### Other material

Swellings have also been recognised in the following specimens from the Koskobilo quarry: *Eomunidopsis navarrensis* (MAB k. 2644, 2794, 2602, 2603, 2924, 2982, 3007, 3191, 3015, 3020, 3302–3309, 3385, 3386), *Eomunidopsis aldoirarensis* (MAB k. 2530, 2981, 3190), *Paragalathea ruizi* (MAB k. 3189, 3310–3312), *Graptocarcinus texanus* (MAB k. 3174), *Faksecarcinus koskobiloensis* (MAB k. 3147, 3149), and *Viaia robusta* (MGSB78508).

Other fossil decapods exhibiting swellings that can also be ascribed to *Kanthyloma crusta* are found in Wienberg Rasmussen *et al.* [18] and references therein, Robins *et al*. [18], and Ceccon & De Angeli [20].

#### Diagnosis

Distinct, typically suboval swelling on convex surface around structure of biogenic origin, not associated with perforations, holes, or other irregularities. Height of swelling typically less than length and width. Orientation of long axis of suboval swelling typically subparallel or at low angle (<45°) to longitudinal axis of host skeleton.

#### Description

Distinct, typically suboval swelling on convex surface around structure of biogenic origin, not associated with perforations, holes, or other irregularities. Height of swelling typically less than length and width. Orientation of long axis of suboval swelling typically subparallel or at low angle (<45°) to longitudinal axis of host skeleton. Swelling not visible from every angle. Swellings do not overlap. Shell outlining swelling generally thin, on the order of millimetres when preserved.

#### Stratigraphic range

?Toarcian–Pleistocene.

#### Remarks

As discussed above, *Kanthyloma crusta* is most likely induced by an isopod parasite. The swelling often increases the maximum width and height of the decapod carapace, whereas maximum carapace length usually is not influenced. The size of the swelling is a few mm up to approximately 16 mm in length [20] and is located in left or right branchial chambers, or both. The swelling may slightly distort ornament by stretching and/or removal of features and distinction between regions.

There is some doubt over the oldest stratigraphic occurrence of the new ichnospecies. Wienberg Rasmussen *et al*. [18] suggested that a swelling of this kind occurred in a specimen of the lobster *Eryma* from Indonesia, but this observation was based on a drawing in Soergel [118]; however, in the text (p. 623), Soergel did refer specifically to it. The youngest example to date from the fossil record is in *Phylira syndactyla* [119] from the Pleistocene of Japan ([18]: pl. 3.10; based on [120]: pl. 1.4). Of note is that Kobayashi *et al*. [120] also showed a conspecific specimen in which both branchial sides appear to be swollen (their pl. 1.19). Holocene decapod fossils with such swellings are to be expected in the future, in view of their occurrence in Pleistocene and modern-day environments.

We are unaware of swellings in places other than the branchial chambers in fossil decapod crustaceans. This is known to be the spot of infestation used by extant members of the bopyrid subfamilies Pseudioninae, Bopyrinae, Argeiinae, Orbioninae and most Keponinae [8], [9]. In modern oceans, some keponins (*Rhopalione* spp.) also infest under the abdomen of pinnotherid crabs, while female athelgines can be found on the dorsal abdomen of paguroids or king crabs [8], [9]. Phyllodurines are known from the ventral surface of abdomina of *Upogebia* (Gebiidea), while members of the Hemiarthrinae have been recorded from the ventral and dorsal abdominal surfaces, or from the lateral sides of carapaces or in the buccal regions of shrimps [8]. Two species within the cryptoniscoid family Entophilidae are endoparasitic, occurring in the visceral cavity of a galatheoid and in the abdomen of a gebiid mud shrimp, respectively [8], [9]. This implies that fossil decapod crustaceans with swellings in other parts of exoskeleton may be found, although abdomina and venters of anomurans and shrimps are rarely represented in the fossil record. Records of isopod-induced swellings on appendages of extant decapods are unknown.

Swellings may also be recognised on well-preserved fossil barnacles. Hosie [121] showed that extant cryptoniscoid isopods are able to induce swellings on stalked cirripedes (his fig. 12A), while Recent cymothooid isopods cause swellings in fish [8]. Such traces are unlikely to fossilise, except under special circumstances. The same applies for isopods that infest non-decapod, softer-bodied crustaceans (see [8]).

## Conclusions

This work provides the first systematic study of isopod infestation for a fossil assemblage of decapod crustaceans. It appears that 4.1% of the brachyuran and anomuran specimens and 33% of the species show a swelling in the mid-Cretaceous (late Albian) fauna from Koskobilo in Spain. Anomurans are more heavily infested than brachyurans, especially the anomuran *Eomunidopsis navarrensis* with 12.1% of the specimens for species with at least 30 specimens. The positive correlation between the number of specimens and the infestation percentage on the species and genus level may suggest host-specificity, but more research is needed to confirm this. Infestation percentages of individual species and across species may have been influenced by a variety of biases including preferential breakage of swellings, differential preservation of taxa, size and final moult-related biases, and the mixture of moults and corpses in this assemblage.

Quantitative decapod infestation patterns through time based on data in the literature and the fauna discussed herein show that the highest number of infested species can be found in the Late Jurassic, also when corrected for the unequal duration of epochs. A peak in the Late Jurassic also shows based on the percentages of infested species per Jurassic and Cretaceous epoch, with Late Jurassic infestation percentages of 11% of all Decapoda, 20% of Brachyura, and 34% of galatheoid Anomura. This supports earlier claims based on much more limited data. The peak infestation in the Late Jurassic and the subsequent drop resulting in lower levels of infestation in the Cretaceous and Cenozoic may have been enhanced by a collecting and reporting bias, but more likely represent a biological signal caused by adaptations increasing resistance to infestation, drops in diversity of infestation-prone decapod groups, copepod (intermediate host)-related changes, and/or changes related to decapod size through time. This type of swellings is now accommodated in a new ichnogenus and -species, *Kanthyloma crusta.*

Much more research can be done to collect and document systematically infestation patterns in other decapod faunas to explain variability within and across genera and families better, refine the temporal trends, gain more insight into the evolutionary understanding of infestation patterns in modern decapods, and to address the various (taphonomic) biases inherent to the fossil record. Therefore, we would like to invite researchers to (continue to) study this phenomenon of infestation in detail.
